# Implications of Protein Interaction in the Speciation of Potential V^IV^O–Pyridinone Drugs

**DOI:** 10.1021/acs.inorgchem.3c01041

**Published:** 2023-05-17

**Authors:** Giarita Ferraro, Maddalena Paolillo, Giuseppe Sciortino, Federico Pisanu, Eugenio Garribba, Antonello Merlino

**Affiliations:** † Department of Chemical Sciences, University of Naples Federico II, Complesso Universitario di Monte Sant’Angelo, Via Cintia, I-80126 Napoli, Italy; ‡ Institute of Chemical Research of Catalonia (ICIQ), The Barcelona Institute of Science and Technology, 43007 Tarragona, Spain; § Dipartimento di Medicina, Chirurgia e Farmacia, Università di Sassari, Viale San Pietro, I-07100 Sassari, Italy

## Abstract

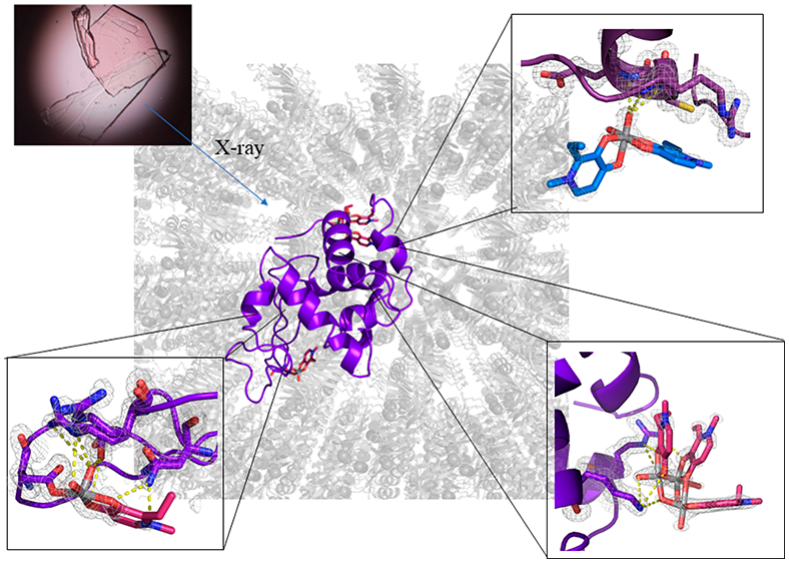

Vanadium complexes (VCs) are promising agents for the treatment, among others, of diabetes and cancer. The development of vanadium-based drugs is mainly limited by a scarce knowledge of the active species in the target organs, which is often determined by the interaction of VCs with biological macromolecules like proteins. Here, we have studied the binding of [V^IV^O(empp)_2_] (where Hempp is 1-methyl-2-ethyl-3-hydroxy-4(1*H*)-pyridinone), an antidiabetic and anticancer VC, with the model protein hen egg white lysozyme (HEWL) by electrospray ionization-mass spectrometry (ESI-MS), electron paramagnetic resonance (EPR), and X-ray crystallography. ESI-MS and EPR techniques reveal that, in aqueous solution, both the species [V^IV^O(empp)_2_] and [V^IV^O(empp)(H_2_O)]^+^, derived from the first one upon the loss of a empp(−) ligand, interact with HEWL. Crystallographic data, collected under different experimental conditions, show covalent binding of [V^IV^O(empp)(H_2_O)]^+^ to the side chain of Asp48, and noncovalent binding of *cis*-[V^IV^O(empp)_2_(H_2_O)], [V^IV^O(empp)(H_2_O)]^+^, [V^IV^O(empp)(H_2_O)_2_]^+^, and of an unusual trinuclear oxidovanadium(V) complex, [V^V^_3_O_6_(empp)_3_(H_2_O)], with accessible sites on the protein surface. The possibility of covalent and noncovalent binding with different strength and of interaction with various sites favor the formation of adducts with the multiple binding of vanadium moieties, allowing the transport in blood and cellular fluids of more than one metal-containing species with a possible amplification of the biological effects.

## Abstract 2

The interaction of [V^IV^O(empp)_2_] (empp(−) = 1-methyl-2-ethyl-3-hydroxy-4(1*H*)-pyridinonato) with hen egg white lysozyme (HEWL) reveals covalent and noncovalent binding to protein of different vanadium-containing fragments: [V^IV^O(empp)(H_2_O)]^+^, [V^IV^O(empp)(H_2_O)_2_]^+^, *cis*-[V^IV^O(empp)_2_(H_2_O)], and even a trinuclear oxidovanadium(V) complex, [V^V^_3_O_6_(empp)_3_(H_2_O)].

## Introduction

After the serendipitous discovery of cisplatin, hundreds of metal complexes (MCs) have been proposed as potential drugs for the treatment of several diseases.^1^ Some MCs, mainly based on Pt, Ag, Au, Ru, Rh, and Ir, have been approved by US Food and Drug Administration (FDA) and/or by European Medicines Agency (EMA) and are now commercially available.^2^ The major problem related to these metals is that they are relatively rare and expensive and are rather toxic for the organism, with a limitation of their use if large doses are required. Essential elements like V, Mn, Cu, and Zn have been tested less than second- and third-row transition metals, despite their high activity; some authors identified the limited industrial development of first-row MCs in medicine in their lability, possibility of hydrolysis, interconversion of geometries and oxidation states, and often in the lack of information on the active species in the organism.^3^

In the series of first-row MCs, vanadium complexes (VCs) have shown high activity and are promising agents for the treatment, among others, of diabetes and several types of cancer.^4^ [V^IV^O(malt)_2_] (bis(maltolato)oxidovanadium(IV) or BMOV), where malt(−) is maltolato ligand,^5^ and [V^IV^O(4,7-dimethyl-1,10-phenanthroline)_2_(SO_4_)] (Metvan)^6,7^ are considered the parent compounds of the class of antidiabetic and antitumor complexes and are frequently used as a benchmark for the development of new vanadium-based agents.

**Scheme 1:**
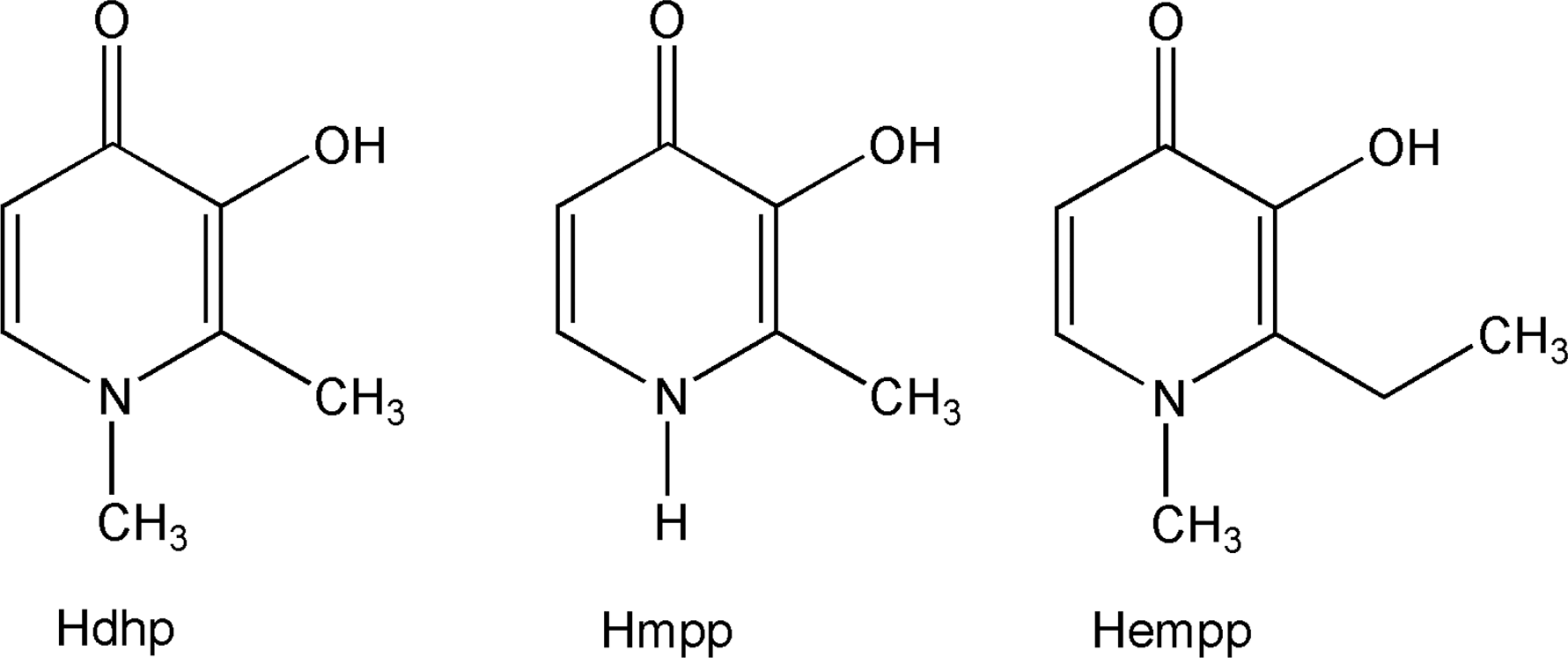
Pyridinone Ligands That Form Pharmacologically Active V^IV^O^2+^ Complexes

Among the most promising VCs, the class of V^IV^O^2+^ complexes formed by pyridinone derivatives is worth being mentioned.^8^ Deferiprone (1,2-dimethyl-3-hydroxy-4(1*H*)-pyridinone; Hdhp, Scheme 1) has long been employed for the treatment of iron overload in *Thalassaemia major*.^9^ More recently, the complex [V^IV^O(dhp)_2_] has been tested as a potential antidiabetic drug.^8^ Interestingly, both [V^IV^O(dhp)_2_] and its derivatives such as [V^IV^O(empp)_2_] and [V^IV^O(mpp)_2_], where Hempp is 1-methyl-2-ethyl-3-hydroxy-4(1*H*)-pyridinone and Hmpp is 2-methyl-3-hydroxy-4(1*H*)-pyridinone (Scheme 1), show high activity. [V^IV^O(empp)_2_] and [V^IV^O(dhp)_2_] are very effective in terms of free fatty acid (FFA) release from isolated rat adipocytes; in particular, the inhibitory effect of [V^IV^O(empp)_2_] on FFA release from rat adipocytes, expressed as half-maximal inhibitory concentration (IC_50_), is 5- and 2-fold higher than V^IV^OSO_4_ and [V^IV^O(dhp)_2_]. Recently, the antiproliferative activity of several V^IV^O-pyridinones against two different malignant melanoma cell lines (A375 and CN-mel) was studied, indicating that they produce apoptosis and cell cycle block.^10^ Therefore, the V^IV^O–pyridinones complexes, having both antidiabetic and anticancer action, can be considered a bridge between these two classes of potential VCs, and, for this reason, some authors suggested their possible application for the treatment of patients suffering from both the diseases.^11^

For VCs with formulation V^IV^OL_2_, where L is a monoanionic bidentate ligand as empp(−), ligand exchange and interaction with bioligands at low vanadium concentration in serum and cytosol are possible. The interacting bioligands may be not only low molecular mass species like amino acids, citrate, lactate, and phosphates, but mainly macromolecules as proteins. Among the proteins, transferrin and albumin in blood serum, hemoglobin in erythrocytes, and phosphatases and ribonucleases in cells are worth of being cited. In various studies the interaction with these proteins has been demonstrated, primarily through electrospray ionization mass spectrometry (ESI-MS), electron paramagnetic resonance (EPR), and computational studies (DFT, QM/MM).^4m,7,12−16^ In general, the most plausible candidates for the vanadium coordination are the residues of Asp, Glu, Asn, Gln, His, and Ser upon replacement of water ligand or the release of one or more ligands.^7,14,15b^

The interaction of proteins with VCs can be covalent or noncovalent. Regarding the first type of binding, the interaction depends on the thermodynamic stability and chemical form of the specific VC: up to four donors could bind V^IV^O^2+^ ion, two donors the fragment V^IV^OL^+^, and one donor the equatorial or axial site of *cis*-octahedral or square pyramidal V^IV^OL_2_ species, respectively.^14,15b^ Until now, structural determinations of the adducts formed by V^IV^O–L moieties with proteins through X-ray diffraction (XRD) are still scarce.^17−20^ In the system with [V^IV^O(malt)_2_], hen egg white lysozyme (HEWL) interacts both noncovalently and covalently with the moieties V^IV^O^2+^ and V^IV^O(malt)^+^, generated upon hydrolysis, and with *cis*-[V^IV^O(malt)_2_(H_2_O)] and *cis*-[V^IV^O(malt)_2_] fragments, the binding occurring with only one donor of the side chain of Glu35, Asp48, Asn65, and Asp87 residues.^20^ In contrast, with V^IV^O(H_2_O)(bipy/phen) the binding of HEWL takes place with the simultaneous coordination of Asn46 and Asp52.^18^ The implications are obvious: the adducts formed after the interaction of a vanadium-based drug with proteins may be the species transported in the biological fluids and/or the active species in the target organs.

In this study, we investigated the interaction of [V^IV^O(empp)_2_] with HEWL through a combined application of ESI-MS and EPR techniques to determine if the binding in solution occurs and through XRD to disclose the three-dimensional structure of the adducts. Due to its high purity, relatively small dimensions, and ease to be studied with ESI-MS, EPR, and X-ray techniques, HEWL has been employed in tens of publications as a model of larger proteins;^16m,21^ as pointed out in the literature, the results obtained with HEWL could give new insights into the binding of biologically active VCs with other proteins, such as albumin or transferrin, disclosing the types of interaction established, covalent or noncovalent, the donors involved in the coordination, and the features and stability of the occupied sites.^21,22^ Concerning the behavior of the system V^IV^O^2+^/Hempp, experimental evidence indicates that [V^IV^O(empp)_2_] is square pyramidal in the solid state,^23,24^ but in solution it is in equilibrium with *cis*-[V^IV^O(empp)_2_(H_2_O)], having the oxido ligand and H_2_O molecule in *cis* position;^24,25^ with varying the pH and decreasing the vanadium concentration, the relative amounts of [V^IV^O(empp)_2_]/*cis*-[V^IV^O(empp)_2_(H_2_O)] and [V^IV^O(empp)(H_2_O)_2_]^+^ species change, allowing several metal moieties to interact with HEWL.

The results of the study may disclose the nature of the interaction of the vanadium-based drugs of pyridinone derivatives and proteins and throw light on the type of the formed adducts that could be the species transported in biological fluids and/or the active species in the target organs.

## Experimental Section

### Materials

Water was deionized through the Millipore Milli-Q Academic system or purchased from Sigma-Aldrich (LC-MS grade). V^IV^OSO_4_**·**3H_2_O, 4-(2-hydroxyethyl)piperazine-1-ethanesulfonic acid (Hepes), sodium formate, sodium acetate, sodium chloride, and succinic acid were Sigma-Aldrich products of the highest grade available and used without further purification. HEWL was purchased from Sigma-Aldrich and used as received. Hempp and [V^IV^O(empp)_2_] were synthesized according to the procedure established previously.^23,24^

### ESI-MS and EPR Measurements

The solutions for ESI-MS studies were prepared in LC-MS grade water dissolving [V^IV^O(empp)_2_] and HEWL to reach a metal-to-protein molar ratio of 2/1 and a metal concentration of 50 μM. The pH of the solution was 4.0 or 7.0, the same used to crystallize the adducts. ESI-MS spectra in positive-ion mode were recorded immediately after the preparation of the solutions with a Q Exactive Plus Hybrid Quadrupole-Orbitrap (Thermo Fisher Scientific) mass spectrometer in the *m*/*z* range 300–4500 and accumulated for at least 5 min to increase the signal-to-noise ratio; the resolution was as high as possible (140 000 in arbitrary units). The experimental parameters were: flow rate of infusion into the ESI chamber 5.00 μL/min; spray voltage 2300 V; capillary temperature 250 °C; sheath gas 10 (arbitrary units); auxiliary gas 3 (arbitrary units); sweep gas 0 (arbitrary units); probe heater temperature 50 °C. ESI-MS spectra were analyzed with Thermo Xcalibur 3.0.63 software (Thermo Fisher Scientific), and the average deconvoluted monoisotopic masses were obtained with the software Unidec 4.4.0.^26^

The EPR spectra were measured in water at pH 7.0 on solutions containing [V^IV^O(empp)_2_] alone or [V^IV^O(empp)_2_] and HEWL at several molar ratios. Hepes (0.1 M) was employed to buffer the solutions. The spectra were recorded at 120 K with an X-band Bruker EMX spectrometer with this instrumental setting: microwave frequency 9.40 GHz; microwave power 20 mW; modulation frequency 100 kHz; modulation amplitude 4.0 G; time constant 81.9 ms; sweep time 335.5 s; resolution 4096 points. To increase the signal-to-noise ratio, signal averaging was used.^27^ In the text the high-field region of the EPR spectra, more sensitive than the low-field one to the identity of the equatorial donors and amount of the species in solution,^28^ is shown; the low-field region is reported in the Supporting Information. The number of scans for the high- and low-field regions of the spectra was 5 or 10 and 5, respectively.

### Crystallization

HEWL crystals were grown by using the hanging drop vapor diffusion method under three different experimental conditions: (i) 1.1 M sodium chloride, 0.1 M sodium acetate at pH 4.0 (structure **A**), (ii) 0.8 M succinic acid at pH 7.0 (structure **B**), and (iii) 2.0 M sodium formate and 0.1 M Hepes at pH 7.5 (structure **C**). These crystals were then exposed to stabilizing solutions containing the mother liquor saturated with [V^IV^O(empp)_2_] for a soaking time in the range of 22–25 days.

### Data Collection and Refinement

Soaked crystals were fished after a few minutes’ incubation in a solution of the reservoir with 25% glycerol and flash-cooled in liquid nitrogen. X-ray diffraction data were collected on three different crystals that diffract X-rays at ultrahigh resolution (1.08–1.10 Å). Data collections were carried out on Beamline XRD2 at Elettra synchrotron (Trieste, Italy), using a wavelength of 1.00 Å and a cold nitrogen stream at 100 K. Data processing and scaling were performed using a Global Phasing autoPROC pipeline.^29^ Data collection statistics are reported in Table S1.

### Structure Solution and Refinement

The structures were phased by molecular replacement, using Protein Data Bank (PDB) entry 193L,^30^ without ligands, as a template, in Phaser software.^31^ Refmac5 was used for refinement.^32^ The presence of vanadium-containing fragments was verified by visual inspection of Fo-Fc and 2Fo-Fc electron density maps in Coot,^33^ while vanadium atom location was confirmed by inspection of anomalous difference electron density maps. Ligand positions were restrained to guide geometry optimization. The final models refine to R-factor and Rfree values within the range of 0.117–0.147 and 0.144–0.189, respectively, with good geometries (Table S1). Figures were drawn using PyMOL.^34^ Coordinates and structure factors of the adducts were deposited in the PDB under the accession codes 8OM8, 8OMS, 8OMT.

## Results and Discussion

### Behavior of [V^IV^O(empp)_2_] in Aqueous Solution

**Figure 1:**
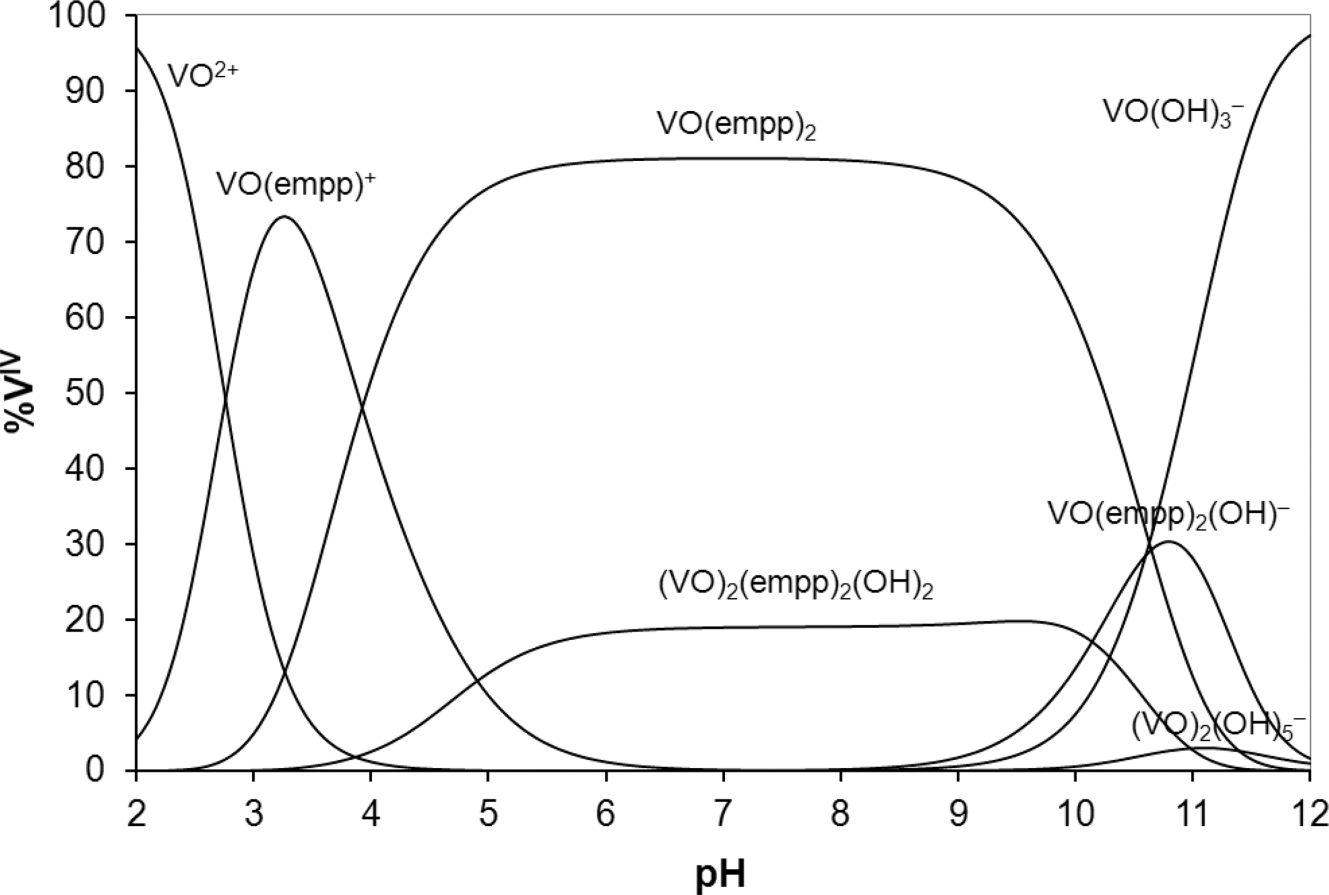
Concentration distribution curves of the species formed as a function of pH in the system V^IV^O^2+^/Hempp 1/2 with vanadium concentration of 50 μM. The water ligands bound to vanadium are omitted for clarity.

[V^IV^O(empp)_2_] has a square pyramidal geometry in the solid state.^23,24^ The thermodynamic stability constants of the complexes formed by V^IV^O^2+^ with empp(−) and its derivatives have been determined.^24^ In the pH range 6–8, with vanadium concentration of 1 mM, the 1:2 V-empp complex overcomes 90%; [V^IV^O(empp)_2_] is in equilibrium with *cis*-[V^IV^O(empp)_2_(H_2_O)], which has the oxido and water ligand in *cis* position.^24^ In aqueous solution, the square pyramidal [V^IV^O(empp)_2_] complex can exist as *SPY*-5–12 and *SPY*-5–13 isomers, while the 1:2 V-empp hexa-coordinated species exist as one of the eight isomers *OC*-6–34-Δ, *OC*-6–34-Λ, *OC*-6–24-Δ, *OC*-6–24-Λ, *OC*-6–32-Δ, *OC*-6–32-Λ, *OC*-6–23-Δ, *OC*-6–23-Λ (Scheme S1), and all the complexes could react, in principle, with a protein. The equatorial water molecule of *cis*-[V^IV^O(empp)_2_(H_2_O)] undergoes deprotonation to give *cis*-[V^IV^O(empp)_2_(OH)]^−^ with a p*K* of 10.65.^24^ With decreasing the concentration of vanadium and pH, the percent amount of [V^IV^O(empp)_2_]/*cis*-[V^IV^O(empp)_2_(H_2_O)] decreases and that of the 1:1 V-empp complex [V^IV^O(empp)(H_2_O)_2_]^+^ increases. *cis*-[V^IV^O(empp)_2_(H_2_O)] and [V^IV^O(empp)(H_2_O)_2_]^+^ could bind to proteins as they stand or as metal moieties *cis-*[V^IV^O(empp)_2_] and [V^IV^O(empp)]^+^ upon the replacement of the equatorial water molecules. The distribution curves of the species formed in the system V^IV^O^2+^/Hempp with molar ratio 1/2 and vanadium concentration 50 μM, used for ESI-MS measurements, are shown in Figure 1; the distribution curves of the same system with a vanadium concentration of 1.0 mM, i.e., the concentration employed for EPR studies, are reported in Figure S1.

### ESI-MS Studies

**Figure 2:**
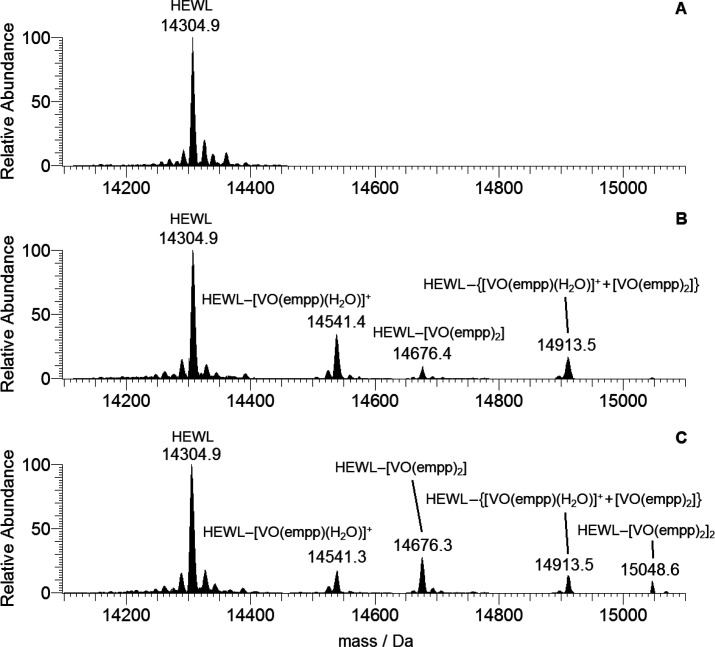
Deconvoluted positive-ion mode spectra recorded on the system containing [V^IV^O(empp)_2_] and HEWL. (A) Free protein at pH 7.0; (B) [V^IV^O(empp)_2_]/HEWL 2/1 at pH 4.0 with a vanadium concentration of 50 μM; (C) [V^IV^O(empp)_2_]/HEWL 2/1 at pH 7.0 with a vanadium concentration of 50 μM.

ESI-MS spectra in the positive-ion mode were measured on fresh solutions containing: (i) HEWL at pH 4.0 and 7.0; (ii) [V^IV^O(empp)_2_]/HEWL with 2/1 molar ratio and metal concentration of 50 μM at pH 4.0 and 7.0, i.e. the two pH values used in the experiments of crystallization (Table S1). The spectra deconvoluted with software Unidec are represented in Figures 2 and S2.

In the spectrum of HEWL, the major peak at 14304.9 Da is surrounded by the signals of the adducts of protein with Na^+^ ion and H_2_O molecules; no peaks were revealed at masses higher than 14400 Da (Figure 2A). The spectra of HEWL recorded in the presence of [V^IV^O(empp)_2_] depend on the pH. With pH = 4.0 three major peaks were detected (Figure 2B): (i) HEWL–[V^IV^O(empp)(H_2_O)]^+^ at 14541.4 Da, with a mass difference relative to free HEWL of +236.6 Da to be compared with expected value of 237.1 Da for the fragment [V^IV^O(empp)(H_2_O)]^+^; (ii) HEWL–[V^IV^O(empp)_2_] at 14676.4 Da, with a mass difference of +371.6 Da with respect to free HEWL to be compared with 371.3 Da for the [V^IV^O(empp)_2_] moiety; (iii) a mixed adduct HEWL–{[V^IV^O(empp)(H_2_O)]^+^ + [V^IV^O(empp)_2_]} at 14915.3 Da, with a difference of +608.7 Da with respect to HEWL to be compared with 608.4 Da expected for {[V^IV^O(empp)(H_2_O)]^+^ + [V^IV^O(empp)_2_]}.

When the pH is increased to pH 7.0 (Figure 2C), the peak of HEWL–[V^IV^O(empp)_2_]_2_ with two [V^IV^O(empp)_2_] moieties interacting with HEWL also appeared; the mass is 15048.6 Da (+743.7 Da with respect to HEWL, to be compared with the mass of 742.6 Da for 2 [V^IV^O(empp)_2_]). Moreover, the intensity of the signals corresponding to the adduct with only one vanadium-containing species (HEWL–[V^IV^O(empp)_2_] at 14676.3 Da) decreases. These results are in agreement with the predictions expected on the basis of the distribution curves of the V^IV^O^2+^ species as a function of pH in Figure 1.

Several observations can be done. (i) The increase of pH favors the interaction with HEWL of [V^IV^O(empp)_2_] with respect to [V^IV^O(empp)]^+^, in agreement with the speciation diagram (Figure 1). (ii) The vanadium-containing fragment with one empp(−) ligand keeps a water molecule coordinated to vanadium (i.e., [V^IV^O(empp)(H_2_O)]^+^), as observed with other pyridinones.^16m^ (iii) The simultaneous binding of vanadium-containing fragments with one or two empp(−) ligands bound to the metal is possible. (iv) The interaction with [V^IV^O(empp)]^+^ is also detected at pH 7.0, even though this species should not exist at such a pH value (see Figure 1); this means that the interaction with protein stabilizes the 1:1 V-empp fragment probably through covalent and/or noncovalent binding. (v) Only the presence of V^IV^O^2+^ adducts is detected, suggesting that the vanadium oxidation state remains +IV under the investigated experimental conditions; it is worth noting that the oxidation of V^IV^ to V^V^ should give adducts with V^V^O_2_ fragment, easily distinguishable from those with V^IV^O^2+^ observed in this study. (vi) Finally, it must be observed that no signals attributable to dinuclear or trinuclear metal species are detected in the ESI-MS spectra.

### EPR Studies

**Figure 3:**
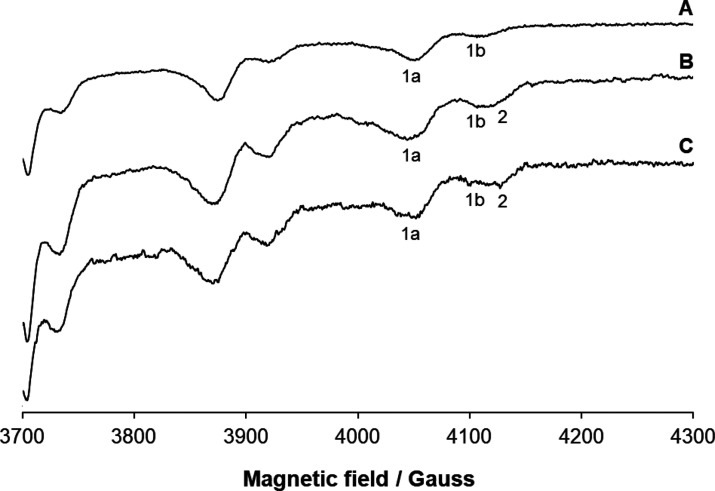
High-field region of the anisotropic X-band EPR spectra recorded at 120 K and pH 7.0 in an aqueous solution containing: (A) [V^IV^O(empp)_2_]; (B) [V^IV^O(empp)_2_]/HEWL 2/1; (C) [V^IV^O(empp)_2_]/HEWL 1/2. Vanadium concentration is 1.0 mM. The *M*_I_ = 7/2 resonances of [V^IV^O(empp)_2_], *cis*-[V^IV^O(empp)_2_], and of the adduct HEWL–[V^IV^O(empp)]^+^ with a covalent binding are indicated with **1a**, **1b**, and **2**, respectively. The interaction of [V^IV^O(empp)_2_] (**1a**) and *cis*-[V^IV^O(empp)_2_] (**1b**) with HEWL could be noncovalent since this does not change the spin Hamiltonian parameters, while that of [V^IV^O(empp)]^+^ could be covalent.

The anisotropic EPR spectra were recorded in the [V^IV^O(empp)_2_]/HEWL 2/1 system at 120 K and pH 7.0. The high-field region is reported in Figure 3, whereas the low-field region is in Figure S3. The spectrum in trace A is recorded in the system obtained dissolving [V^IV^O(empp)_2_] in water with Hepes as the buffer. The two species detected are [V^IV^O(empp)_2_] (indicated by **1a** in Figure 3) with a value of *g* factor and hyperfine coupling constant on *z* axis (*g*_*z*_ and *A*_*z*_) of 1.953 and 158 × 10^–4^ cm^–1^, respectively, and the complex in equilibrium with it, *cis*-[V^IV^O(empp)_2_(H_2_O)] (**1b**) with *g*_*z*_ = 1.943 and *A*_*z*_ 168 × 10^–4^ cm^–1^. These values are in agreement with those reported in the literature.^24^ The signals detected in the system containing HEWL (traces B and C) were assigned to the same species, [V^IV^O(empp)_2_] (**1a**) and *cis*-[V^IV^O(empp)_2_(H_2_O)] (**1b**). This means that the two 1:2 V-empp species keep their structure and only a noncovalent interaction with HEWL—which does not change spin Hamiltonian parameters—could take place. The absence of other signals in the region 3960–4100 G suggests that the formation of adducts with a covalent bond between the [V^IV^O(empp)_2_] moiety and a side chain of HEWL, in principle possible in the axial accessible site of **1a** or in the equatorial site of **1b**, does not occur. This is in contrast with what was observed when HEWL reacts with *cis*-[V^IV^O(malt)_2_], where the formation of a V^IV^–O(Asn) bond has been demonstrated with XRD.^20^ The larger steric hindrance and/or lower stabilization through secondary interactions such as hydrogen bonds or van der Waals contacts for [V^IV^O(empp)_2_] may account for these experimental findings. However, the resonances around at 4130 G indicate the presence of another species, with *g*_*z*_ ≈ 1.942 and *A*_*z*_ ≈ 173 × 10^–4^ cm^–1^ (**2** in Figure 3). This is probably an adduct formed by a 1:1 V-empp species, [V^IV^O(empp)(H_2_O)]^+^, with HEWL.

The formation of simultaneous coordination of [V^IV^O(empp)_2_] and [V^IV^O(empp)(H_2_O)]^+^ cannot be excluded. Therefore, overall, the EPR results agree well with ESI-MS measurements that indicate the formation of HEWL–[V^IV^O(empp)_2_] and HEWL–[V^IV^O(empp)(H_2_O)]^+^ adducts, plus HEWL–{[V^IV^O(empp)(H_2_O)]^+^ + [V^IV^O(empp)_2_]} (see Figure 2).

### Crystallographic Studies

**Figure 4:**
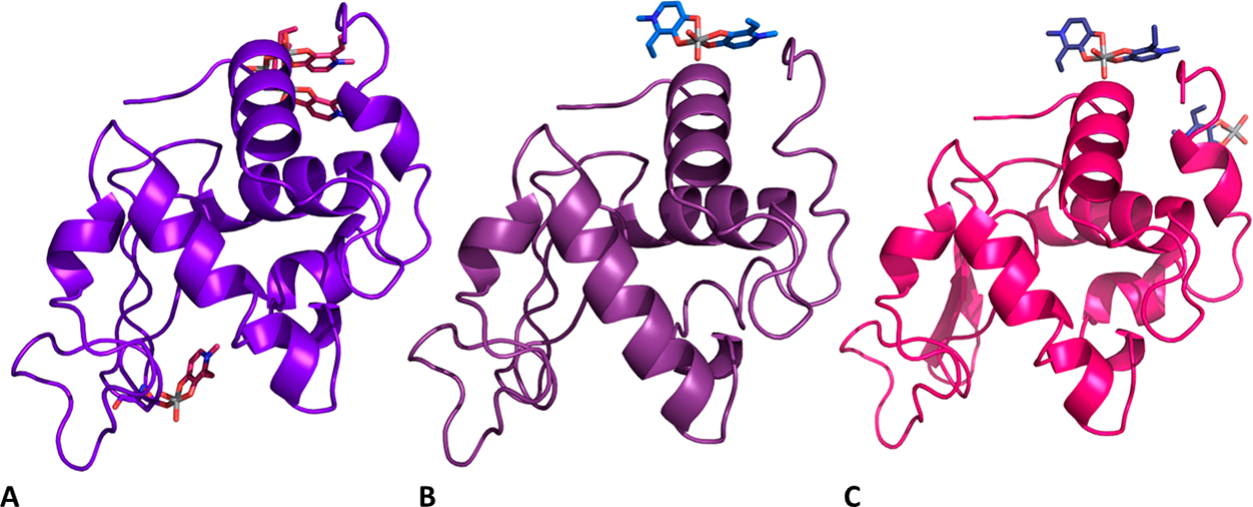
Overall structures of the adducts formed upon reaction of [V^IV^O(empp)_2_] with HEWL under different experimental conditions: (A) structure **A**, derived from a crystal grown in 1.1 M sodium chloride, 0.1 M sodium acetate at pH 4.0; (B) structure **B**, derived from a crystal grown in 0.8 M succinic acid at pH 7.0; (C) structure **C**, derived from a crystal grown in 2.0 M sodium formate, 0.1 M Hepes at pH 7.5. Vanadium atoms are in green. Coordinates and structure factors were deposited in the PDB under the accession codes 8OM8, 8OMS, 8OMT.

**Figure 5:**
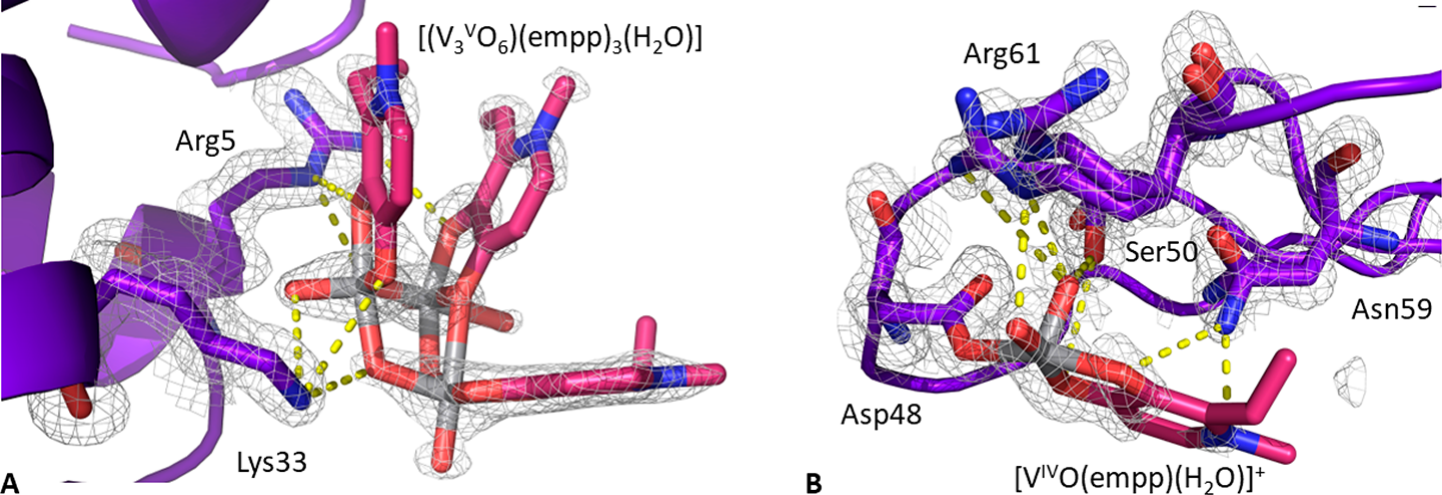
Vanadium binding sites in structure **A**: (A) noncovalent binding of [(V^V^_3_O_6_)(empp)_3_(H_2_O)]; (B) covalent coordination of [V^IV^O(empp)(H_2_O)]^+^ to the side chain of Asp48. 2Fo-Fc electron density maps are reported at 1.0 σ level in gray.

To structurally characterize the interaction of HEWL with [V^IV^O(empp)_2_], crystals of metal-free protein, grown under three different conditions, were exposed for 22–25 days to stabilizing solutions containing the mother liquors saturated with the VC. All the crystals belong to the space group *P*4_3_2_1_2 and present one single protein molecule in the asymmetric unit. In structure **A** (Figure 4A), obtained analyzing data collected on a crystal grown in 1.1 M sodium chloride, 0.1 M sodium acetate at pH 4.0, the noncovalent binding of a trinuclear oxidovanadium(V) complex on the protein surface was observed (this is illustrated in Figure 5A), together with the covalent binding of [V^IV^O(empp)(H_2_O)]^+^ to the side chain of Asp48 (illustrated in Figure 5B).

2Fo-Fc electron density map at these sites, reported at 1.0 σ in Figure 5A,B, indicates a clear definition of the metal geometry and its ligands. Refinements suggest an occupancy value of 0.6 for these two vanadium-containing fragments (Table S1). The metal cluster found on HEWL surface consists of a cyclic trinuclear oxidovanadium(V) complex, [V^V^_3_O_6_(empp)_3_(H_2_O)]. An analogous species, [V^V^_3_O_6_(dhp)_3_(H_2_O)], was isolated from the reaction of Hdhp, the methyl derivative of Hempp, KOH, and sodium metavanadate at pH 4.5,^35^ the same as that used in our experiment, and was characterized in aqueous solution in the system V^V^/Hdhp in the pH range 2.0–5.0.^36^ This means that the oxidation of V^IV^ to V^V^ takes place under the investigated experimental conditions, with the consequent formation of the trinuclear unit, probably favored by the relatively high local vanadium concentration reached during the soaking experiments and by crystal lattice environment, which can serve as a chemical reaction vessel. Notably, the results obtained in a simple inorganic system have been replicated in a complex biological system.

As observed for [V^V^_3_O_6_(dhp)_3_(H_2_O)] structure solved by Avecilla et al.,^35^ one out of the three V^V^ atoms is penta-coordinated with a distorted square pyramidal geometry, while the other two are hexa-coordinated with a distorted octahedral arrangement. Each metal center is bound by one oxido group, two O bridging ligands, and two empp(−) oxygen atoms. One of the two octahedral vanadium atoms is also coordinated to a third oxygen from a second empp(−) anion, this ligand acting as a bridge between the two hexa-coordinated V^V^ centers; the second octahedral V^V^ completes its coordination sphere with a water molecule. Two of the three empp(−) ligands show an (equatorial-equatorial) arrangement, with two oxygens in *cis* position relative to the V=O bond, while the third one occupies an equatorial and an axial position. The V=O bond distances have similar values (1.62–1.65 Å) that are all compatible with those experimentally observed in the similar structure with dhp.^35^ As expected, the V–OH_2_ distance is slightly longer (2.03 Å). The three V–O–V bond angles in the cyclic framework, involving single bridging oxygen atoms, are very similar, with values close to 120°. A detailed comparison of selected bond lengths and angles observed in [V^V^_3_O_6_(empp)_3_(H_2_O)] and in [V^V^_3_O_6_(dhp)_3_(H_2_O)] is reported in Table S2, while the structure of trinuclear cluster formed by empp(−) in the system with HEWL is represented in Figure S4. The binding of [V^V^_3_O_6_(empp)_3_(H_2_O)] to HEWL is stabilized by stacking interactions of the two empp(−) ligands, which are almost parallel to each other, with the side chain of Trp123 and by hydrogen bonds of the bridging oxygens with the side chains of Arg5 and Lys33 (Figure 5A); [(V^V^_3_O_6_)(empp)_3_(H_2_O)] is also hydrogen-bonded to water molecules and to the side chains of Arg73 and Asp101 of a symmetry-related molecule.

Concerning the binding of the [V^IV^O(empp)(H_2_O)]^+^ fragment in structure **A**, V^IV^ is coordinated to the side chain of Asp48 which—in turn—is held in its position by hydrogen bonds formed with Ser50, Asn59, and Arg61 side chains (represented in Figure 5B), with water molecules, and also with Gln121 and Asp125 side chains from a symmetry-related molecule. This agrees with ESI-MS and EPR results that indicate the formation of the adduct HEWL–[V^IV^O(empp)(H_2_O)]^+^ (Figures 2 and 3).

**Figure 6:**
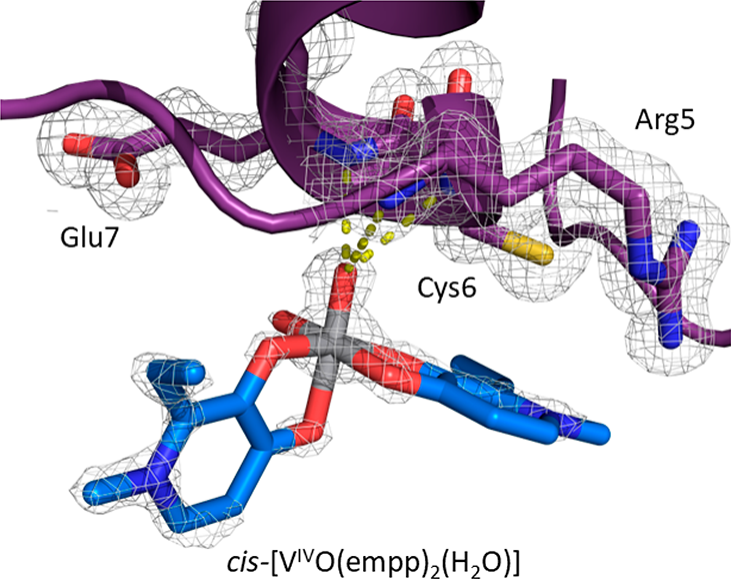
Vanadium binding site in structure **B**: noncovalent binding of *cis*-[V^IV^O(empp)_2_(H_2_O)]. 2Fo-Fc electron density maps are reported at 1.0 σ level in gray.

In structure **B** (Figure 4B), derived from crystals grown in 0.8 M succinic acid at pH 7.0, noncovalent binding of *cis*-[V^IV^O(empp)_2_(H_2_O)] on the protein surface was found (occupancy = 0.40). This vanadium-containing fragment is noncovalently bound to HEWL through hydrogen bonds with N main chain atoms of Arg5, Cys6, and Glu7 (Figure 6), water molecules, and Arg14 side chain from a symmetry-related molecule. The noncovalent binding to HEWL of *cis*-[V^IV^O(empp)_2_(H_2_O)] was also suggested by EPR spectroscopy (resonances indicated by **1b** in Figure 3).

**Figure 7:**
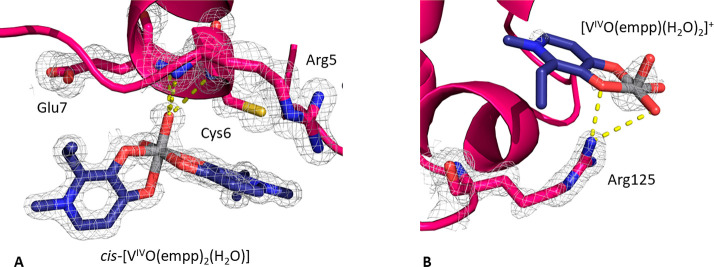
Vanadium binding site in structure **C**: (A) noncovalent binding of *cis*-[V^IV^O(empp)_2_(H_2_O)]; (B) noncovalent binding of [V^IV^O(empp)(H_2_O)_2_]^+^. 2Fo-Fc electron density maps are reported at 1.0 σ level in gray.

The structure **C** was derived from a crystal grown in 2.0 M sodium formate and 0.1 M Hepes at pH 7.5 (Figure 4C). In this structure, the noncovalent binding of *cis*-[V^IV^O(empp)_2_(H_2_O)] fragment (occupancy = 0.80) was observed (Figure 7A). However, under these conditions, a [V^IV^O(empp)(H_2_O)_2_]^+^ species (occupancy = 0.40), noncovalently bound, was also found; this additional vanadium-containing fragment forms hydrogen bonds with Arg125 side chain and water molecules (Figure 7B). The simultaneous binding of *cis*-[V^IV^O(empp)_2_(H_2_O)] and [V^IV^O(empp)(H_2_O)]^+^ to HEWL was also disclosed by ESI-MS measurements presented in Figure 2.

In conclusion, crystallographic data provide insights into the role of HEWL interaction in the speciation of [V^IV^O(empp)_2_]. Under the investigated experimental conditions, [V^IV^O(empp)(H_2_O)]^+^, [V^IV^O(empp)(H_2_O)_2_]^+^, *cis*-[V^IV^O(empp)_2_(H_2_O)] derived from the reaction [V^IV^O(empp)_2_] + H_2_O ⇄ *cis*-[V^IV^O(empp)_2_(H_2_O)] and the trinuclear V^V^ species [V^V^_3_O_6_(empp)_3_(H_2_O)] can bind the protein. Notably, even though in aqueous solution the two species [V^IV^O(empp)_2_] and *cis*-[V^IV^O(empp)_2_(H_2_O)] are in equilibrium between each other, no binding of the square pyramidal species [V^IV^O(empp)_2_] is detected. For *cis*-[V^IV^O(empp)_2_(H_2_O)] only the isomer with two equatorial phenolate-O is revealed; furthermore, only the enantiomer *OC*-6–24-Λ (see Scheme S1) interacts with the protein. Finally, as suggested by ESI-MS results, the simultaneous binding of more than one vanadium-containing moiety, i.e., the 1:1 and 1:2 V-empp species, is possible. This could be important in the transport and mechanism of action of this class of potential metal drugs.

### Comparison with the Behavior of [V^IV^O(malt)_2_] and Other [V^IV^OL_2_] Potential Drugs

The comparison between the binding modes to HEWL of [V^IV^O(empp)_2_] and other vanadium-based potential drugs with formulation [V^IV^OL_2_] reveals analogies and differences that can be related to the ligand structure, stability of the V^IV^O^2+^ species, possibility of formation of covalent and/or noncovalent bonds, and stabilization of the adducts through secondary interactions like hydrogen and van der Waals contacts. Such features can affect the transport in biological fluids and the interaction with the targets in the organism and, ultimately, the pharmacological action.

The behavior of the [V^IV^O(malt)_2_]/HEWL system has been recently investigated through the combined application of ESI-MS, EPR, and X-ray crystallography techniques.^20^ Three sites with the multiple binding of different vanadium-containing species were identified: in particular, the noncovalent binding of *cis*-[V^IV^O(malt)_2_(H_2_O)] and [V^IV^O(malt)(H_2_O)_3_]^+^, and—also—the covalent binding of [V^IV^O(H_2_O)_3–4_]^2+^ and *cis*-[V^IV^O(malt)_2_] moieties to the side chains of Glu35, Asp48, Asn65, Asp87, and Asp119 and to the C-terminal carboxylate were demonstrated.^20^

**Figure 8:**
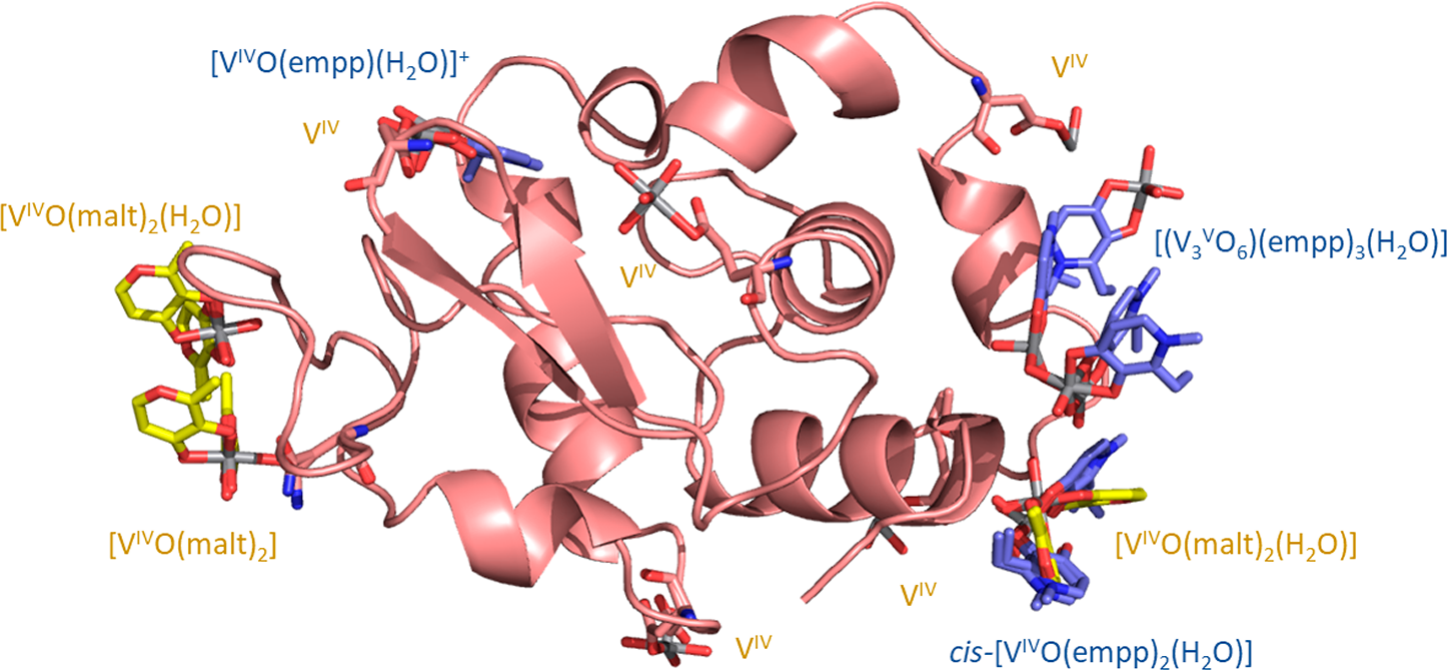
Superimposition of the structures of the adducts formed by HEWL with [V^IV^O(malt)_2_] and [V^IV^O(empp)_2_]. [V^IV^O(malt)_2_] fragments and vanadium atoms derived from [V^IV^O(malt)_2_] are in yellow, [V^IV^O(empp)_2_] fragments and [(V^V^_3_O_6_)(empp)_3_(H_2_O)] are in blue.

The comparison with [V^IV^O(empp)_2_]/HEWL allows one to find differences and similarities of the two systems, described graphically in Figure 8.

(1) While *cis*-[V^IV^O(malt)_2_(H_2_O)] gives both noncovalent and covalent binding through the replacement of the water molecule by Asn65 side chain,^20^ in the structures here obtained, only the noncovalent interaction of *cis*-[V^IV^O(empp)_2_(H_2_O)] is observed.
(2) In the [V^IV^O(malt)_2_]/HEWL system, covalent or noncovalent interaction of various isomers—with two equatorial phenolato-O^–^ or two equatorial keto-O—is found; moreover, three enantiomers (*OC*-6–24-Λ, *OC*-6–24-Δ, and *OC*-6–34-Δ) are present in the adducts.^20^ In contrast, with empp(−) only the isomer with two equatorial phenolato-O^–^ and only the enantiomer *OC*-6–24-Λ binds to HEWL.
(3) The interaction with [V^IV^O(empp)_2_] is favored in the two systems with pH 7.0 and 7.5 (structures **B** and **C**), when this species reaches its maximum amount as suggested by the concentration distribution curves in Figure 1.
(4) Despite the 1:1 V-empp species should not exist at pH 7.0, the adduct HEWL–[V^IV^O(empp)(H_2_O)]^+^ is detected in solution with ESI-MS and EPR, and both [V^IV^O(empp)(H_2_O)]^+^ and [V^IV^O(empp)(H_2_O)_2_]^+^ are revealed with XRD in the solid state adducts. This means that the interaction with the protein stabilizes the 1:1 V-empp species, allowing them to bind through covalent or noncovalent binding.
(5) The interaction of 1:1 V-empp species with HEWL can be covalent or noncovalent. It is covalent at pH 4.5 and noncovalent at pH 7.0. In the [V^IV^O(malt)_2_]/HEWL system, instead, only a noncovalent interaction of [V^IV^O(malt)(H_2_O)_3_]^+^ was ascertained.^20^
(6) The oxidation of V^IV^ to V^V^ with formation of the trinuclear species [V^V^_3_O_6_(empp)_3_(H_2_O)] is observed in the [V^IV^O(empp)_2_]/HEWL system. With maltolato, no similar adducts were isolated, and no clues of a possible oxidation of vanadium were found.^20^
(7) While in the [V^IV^O(malt)_2_]/HEWL system the formation of adducts with the ion V^IV^O^2+^ was demonstrated,^20^ with [V^IV^O(empp)_2_] this does not occur. This could be attributable to the larger thermodynamic stability of the V^IV^O^2+^ complexes with empp(−). From the distribution curves of the species formed as a function of pH, V^IV^O^2+^ does not survive in solution for pH higher than 4.0, both with vanadium concentration of 50 μM and 1 mM (Figures 1 and S1).
(8) V binding sites in the [V^IV^O(malt)_2_]/HEWL and [V^IV^O(empp)_2_]/HEWL systems are distinct (Table S3), but there are also common features. A conserved vanadium binding site was found close to Asp48 side chain, that binds [V^IV^O(empp)(H_2_O)]^+^ (this study) and [V^IV^O(H_2_O)_3_]^2+^ in the system with maltolato ligand.^20^ A site for noncovalent binding, close to main chain atoms of Arg5, Cys6, and Glu7, is also conserved.

A final comment concerns the binding sites of HEWL with other [V^IV^OL_2_] potential drugs, such as [V^IV^O(pic)_2_(H_2_O)],^17^ [V^IV^O(phen)_2_],^18^ and [V^IV^O(bipy)_2_]^18^ (Table S3). It is noteworthy that the comparison of these systems with [V^IV^O(empp)_2_]/HEWL reveals that the vanadium binding sites that have been found up to now are different and that the preferential binding is covalent with the involvement of Asn and Asp residues.

## Conclusions

Here we have studied the reactivity of the potential drug [V^IV^O(empp)_2_] with HEWL, using a combination of physicochemical techniques, which include mass spectrometry, electron paramagnetic resonance, and macromolecule X-ray crystallography. The ligand empp(−) belongs to the family of pyridinones that form with V^IV^ promising agents for the treatment of diabetes and cancer. In aqueous solution the results reveal that (i) the protein interacts with [V^IV^O(empp)_2_] and [V^IV^O(empp)(H_2_O)]^+^; (ii) both covalent and noncovalent binding of the vanadium species to HEWL can occur; (iii) simultaneous interaction of more than one vanadium-containing fragment is possible.

Three X-ray structures of HEWL in the presence of [V^IV^O(empp)_2_] were solved under different experimental conditions. Vanadium-containing fragment binding does not alter the overall conformation of the protein. Covalent binding of [V^IV^O(empp)(H_2_O)]^+^ and noncovalent binding of *cis*-[V^IV^O(empp)_2_(H_2_O)], [V^IV^O(empp)(H_2_O)]^+^, and [V^IV^O(empp)(H_2_O)_2_]^+^ to the protein was demonstrated, in agreement with spectrometric/spectroscopic data. Side chain of Asp48 is involved in the coordination of the moiety [V^IV^O(empp)(H_2_O)]^+^. Notably, the X-ray structure solved from crystals grown at acidic pH also shows noncovalent binding to HEWL of a trinuclear oxidovanadium(V) compound; to the best of our knowledge, this is an unprecedented example of a protein adduct with a trinuclear oxidovanadium compound. The formation of the trinuclear complex is likely favored by the experimental conditions needed to obtain crystals of the protein and, in particular, by the acidic pH and high vanadium concentration, but it seems plausible that a role in the formation of the species could be played by the crystal lattice environment, which can serve as a chemical reaction vessel. Differences in the results obtained by ESI-MS and EPR—which, for example, do not show the oxidation of V^IV^ to V^V^—and XRD can be easily explained considering the differences in the used experimental conditions, as the long soaking time. Overall, our results confirm that studies on the interaction of VCs with proteins are needed to discover the biologically active species that form in the cellular *milieu*. Indeed, the presence of proteins can alter the speciation of active V^IV^OL_2_ compounds, which can lose their L ligands before and upon interaction with these biological macromolecules. Such an interaction could favor the formation of fragments with water molecules replacing the carrier ligand, the oxidation of vanadium, and/or, depending on the metal concentration and experimental conditions, the formation of oligomeric species.

Comparison between the here-reported and the previously published structural results on adducts formed upon reaction of pharmacologically active [V^IV^OL_2_] compounds with HEWL indicates that (i) several accessible vanadium binding sites exist on HEWL surface, (ii) the number and the type of vanadium binding sites is different in the characterized adducts, (iii) the occupancies of vanadium-containing fragments can significantly vary (from 0.25 to 0.90), and (iv) the binding can be covalent or noncovalent. These data suggest that the vanadium moieties distribute among various sites with comparable energy, to form adducts stabilized by hydrogen bonds and van der Waals contacts, and that the specific experimental conditions could determine the species isolated in the solid state and characterized by XRD analysis. In other words, one can imagine that, in solution, a vanadium species goes from a bound to an unbound state in a complex process in which the interaction sites change with time but the binding with protein remains. The possibility of interaction with different sites and of covalent and noncovalent binding with variable strength would favor the formation of adducts with the multiple binding of vanadium moieties, allowing the transport in blood and cellular fluids of more than one vanadium-containing species with a significant amplification of the biological metal effects.

Therefore, the data collected in the literature up to now on the binding VCs–protein would suggest that the different action of potential vanadium drugs could be explained, at least in part, with the different interaction with the macromolecules, even though other results in the next future are necessary to confirm these conclusions. Overall, these observations could open new *scenarios* in the description of the transport and mechanism of action not only of vanadium but also, more in general, of metal-based drugs, promoting the development of new compounds as potential therapeutic agents.

## Supporting Information Available

Tables with data collection and refinement statistics for (V complexes)–HEWL adducts in structures **A**, **B**, and **C** (Table S1), selected bond lengths and angles for single crystals of [V^V^_3_O_6_(dhp)_3_(H_2_O)] and of the adduct [V^V^_3_O_6_(empp)_3_(H_2_O)]–HEWL (Table S2), vanadium binding sites found in the crystal structures of HEWL treated with potential [V^IV^OL_2_] drugs (Table S3), figures with isomers/enantiomers of [V^IV^O(empp)_2_(H_2_O)] (Scheme S1), concentration distribution curves of V^IV^O^2+^/Hempp system (Figure S1), deconvoluted ESI-MS spectrum of HEWL at pH 4.0 (Figure S2), low-field region of the EPR spectra (Figure S3), structure of the [V^V^_3_O_6_(empp)_3_(H_2_O)] species bound to HEWL (Figure S4) (PDF)
