# Supplementary material for: Implications of Protein Interaction in the Speciation of Potential VIVO–Pyridinone Drugs
Source: Inorg Chem. 2023 May 17;62(21):8407–17. doi: 10.1021/acs.inorgchem.3c01041 (PMC10230503; doi:10.1021/acs.inorgchem.3c01041)
Supplement: Supplementary file 1 — ic3c01041_si_001.pdf [file ic3c01041_si_001.pdf]

SUPPORTING INFORMATION

Implications of protein interaction in the speciation of potential $V^{IV}O$–pyridinone drugs

Giarita Ferraro,[a] Maddalena Paolillo,[a] Giuseppe Sciortino,[b] Federico Pisanu,[c] Eugenio Garribba,[c,] Antonello Merlino,[a,]**

[a] Department of Chemical Sciences, University of Naples Federico II, Complesso Universitario di Monte Sant'Angelo, Via Cintia, I-80126, Napoli, Italy

[b] Institute of Chemical Research of Catalonia (ICIQ), The Barcelona Institute of Science and Technology, 43007 Tarragona, Spain

[c] Dipartimento di Medicina, Chirurgia e Farmacia, Università di Sassari, Viale San Pietro, I-07100 Sassari, Italy

Corresponding authors. E-mail: garribba@uniss.it (E.G.); antonello.merlino@unina.it (A.M.).

Table S1. Data collection and refinement statistics for VCs–HEWL adducts in structures **A**, **B** and **C**.

	Structure **A**	Structure **B**	Structure **C**
Crystallization conditions	*1.1 M sodium chloride, 0.1 M sodium acetate at pH 4.0*	*0.8 M succinic acid at pH 7.0*	*2.0 M sodium formate and 0.1 M Hepes at pH 7.5*
Space group	$P4_3\ 2_1\ 2$	$P4_3\ 2_1\ 2$	$P4_3\ 2_1\ 2$
a (Å)	78.41	77.59	77.13
b (Å)	78.41	77.59	77.13
c (Å)	37.02	37.58	37.26
α/β/γ (°)	90.0/90.0/90.0	90.00/90.00/90.00	90.0/90.0/90.0
Molecules im the asymmetric unit	1	1	1
Resolution range (Å)	55.44-1.08 (1.10-1.08)	38.80-1.10 (1.22-1.10)	38.56-1.08 (1.10-1.08)
Observations	1139882 (43874)	814761 (18987)	701425 (3107)
Unique reflections	50384 (2483)	46373 (2164)	40727 (820)
Completeness (%)	100.0 (100.0)	99.4 (95.0)	83.9 (34.0)
Redundancy	22.6 (17.7)	17.6 (8.8)	17.2 (3.8)
Rmerge (%)	0.045 (1.409)	0.051 (0.645)	0.043 (0.404)
Average I/σ(I)	33.2 (2.2)	28.8 (2.9)	36.5 (2.7)
$CC_{1/2}$	1.000 (0.744)	0.999 (0.867)	1.000 (0.829)
Anom. completeness (%)	99.9 (100.0)	99.1 (91.1)	83.8 (32.0)
Anom. Multiplicity	12.0 (9.2)	9.3 (4.8)	9.2 (2.1)
Resolution (Å)	55.26-1.08	38.80-1.10	38.56-1.10
N° reflections	43357	44373	38056
N° reflections in working set	1803	3090	1319
Rfactor/Rfree	0.147/0.189	0.144/0.167	0.116/0.144
N° non-H atoms in the refinement	1311	1290	1317
Estimated occupancy of $[(V^{V}{}_3O_6)(empp)_3(H_2O)]$ in structure **A**	0.60	–	–
Estimated occupancy of $[V^{IV}O(empp)(H_2O)]^+$ in structure **A**	0.60	–	–
B-factor overall (Å^2)	20.12	15.69	15.97
B-factor of $[(V^{V}{}_3O_6)(empp)_3(H_2O)]$ in structure **A** (Å^2)	26.3 ± 2.7	–	–
B-factor of	23.24	–	–

$[VO(empp)(H_2O)]^+$ in structure **A** (Å^2)			
Estimated occupancy of *cis*-$[V^{IV}O(empp)_2(H_2O)]$ in structure **B**	–	0.40	–
B-factor of *cis*-$[VO(empp)_2(H_2O)]$ in structure **B** (Å^2)	–	17.14	–
Estimated occupancy of *cis*-$[V^{IV}O(empp)_2(H_2O)]$ in structure **C**	–	–	0.80
Estimated occupancy of $[V^{IV}O(empp)(H_2O)_2]^+$ in structure **C**	–	–	0.40
B-factor of *cis*-$[V^{IV}O(empp)_2(H_2O)]$ in structure **C** (Å^2)	–	–	14.51
B-factor of $[V^{IV}O(empp)(H_2O)_2]^+$ in structure **C** (Å^2)	–	–	37.39
Most favoured/ Additional allowed	94.06%/5.94%	95.15%/4.85%	95.33%/4.67%
Outliers	0	0	0
Rmsd bonds (Å)	1203	1153	1156
Rmsd angles (°)	1648	1576	1583
PDB code	8OM8	8OMS	8OMT

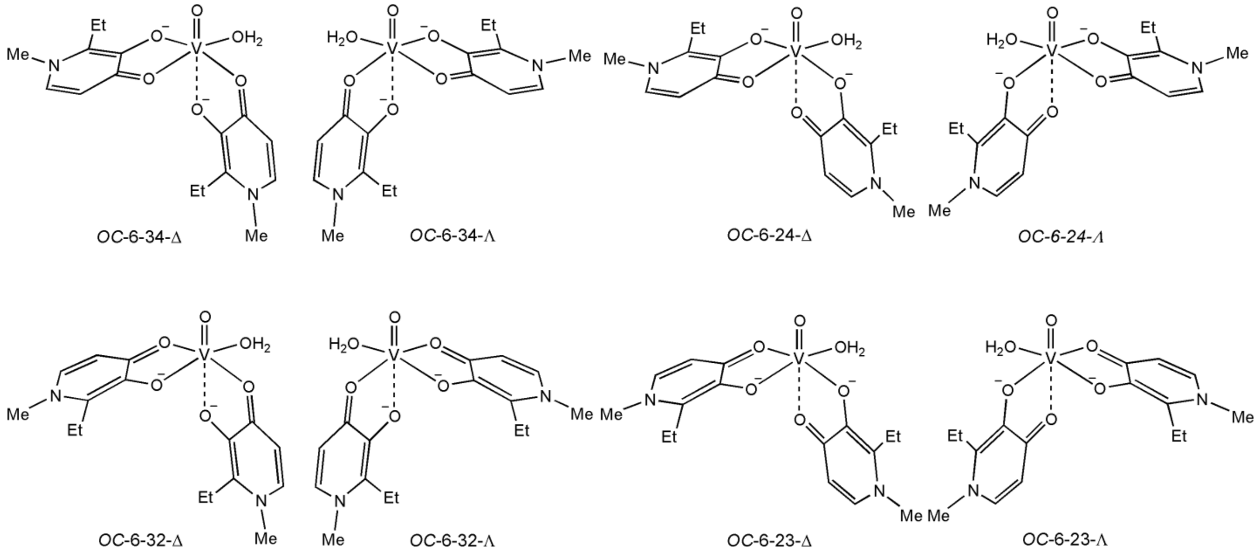

Scheme S1. Isomers/enantiomers of *cis*-[$V^{IV}O(empp)_2(H_2O)$] complex formed after the dissolution of [$V^{IV}O(empp)_2$] in aqueous solution.

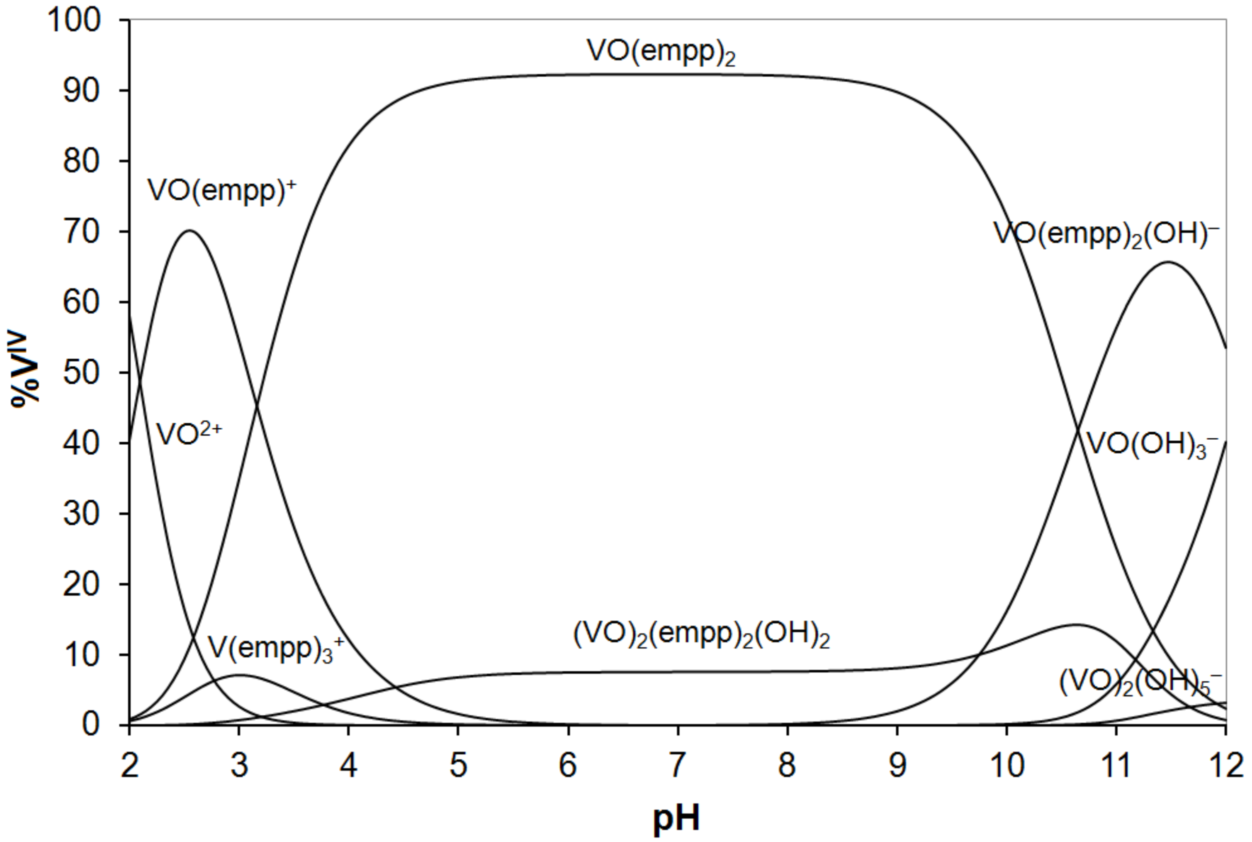

Figure S1. Concentration distribution curves of the species formed as a function of pH in the system $V^{IV}O^{2+}$/Hempp 1/2 with vanadium concentration of 1.0 mM. The water ligands bound to vanadium are omitted for clarity.

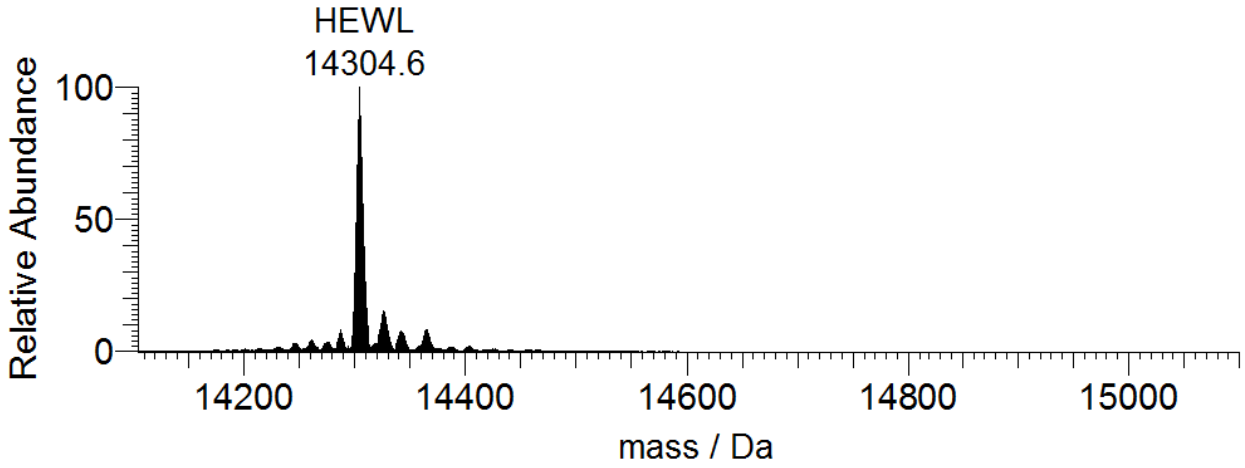

Figure S2. Deconvoluted ESI-MS spectrum of HEWL (pH 4.0, protein concentration 25 μM).

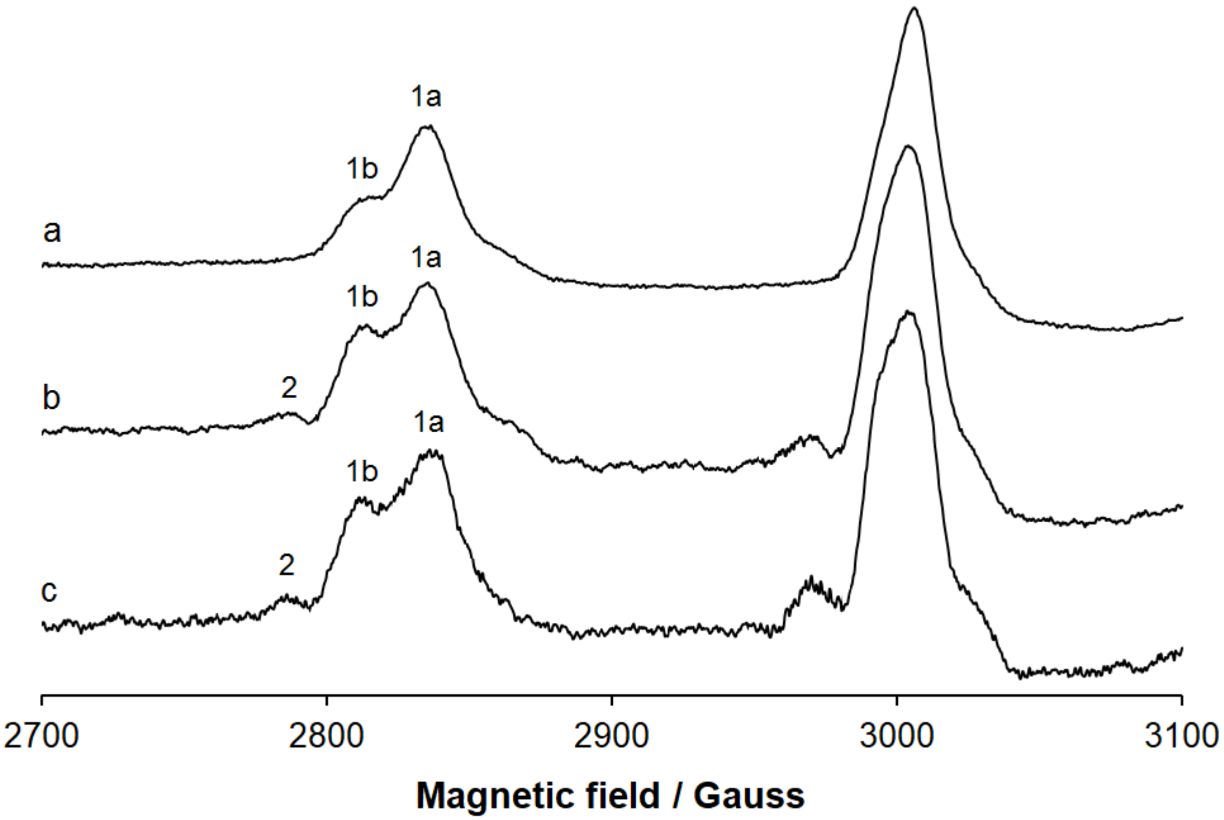

Figure S3. Low-field region of the anisotropic X-band EPR spectra recorded at 120 K and pH 7.0 in an aqueous solution containing: A) [$V^{IV}O(empp)_2$]; B) [$V^{IV}O(empp)_2$]/HEWL 2/1; C) [$V^{IV}O(empp)_2$]/HEWL 1/2. V concentration is 1.0 mM. The M_I = -7/2 resonances of [$V^{IV}O(empp)_2$], *cis*-[$V^{IV}O(empp)_2$] and of the adduct HEWL–$V^{IV}O$(empp) are indicated with **1a**, **1b** and **2**, respectively. The interaction of [$V^{IV}O(empp)_2$] (**1a**) and *cis*-[$V^{IV}O(empp)_2$] (**1b**) with HEWL could be non-covalent since this does not change spin Hamiltonian parameters, while that of [$V^{IV}O(empp)]^+$ could be covalent.

Table S2. Selected bond lengths (Å) and angles (°) for [$V^V{}_3O_6$(empp)$_3$(H_2O)] in its adduct with HEWL, and comparison with the values found in single crystals of [$V^V{}_3O_6$(dhp)$_3$(H_2O)].[a]

	[$V^V{}_3O_6$(empp)$_3$(H_2O)]	[$V^V{}_3O_6$(dhp)$_3$(H_2O)] [b]
Bond	Bond lengths (Å)	Bond lengths (Å)
V(1)-O(1)	1.65	1.599(3)
V(1)-O(6)	1.76	1.794(3)
V(1)-O(4)	1.99	1.817(3)
V(1)-O(7)	1.94	1.980(4)
V(1)-O(8)	1.79	2.008(4)
V(1)-O(12)	2.29	2.450(3)
V(2)-O(2)	1.62	1.613(4)
V(2)-O(4)	1.93	1.795(3)
V(2)-O(5)	1.81	1.824(4)
V(2)-O(9)	1.97	1.962(3)
V(2)-O(10)	1.75	2.004(4)
V(3)-O(3)	1.62	1.609(4)
V(3)-O(5)	1.82	1.813(3)
V(3)-O(6)	1.98	1.857(3)
V(3)-O(11)	1.81	1.909(3)
V(3)-O(12)	2.05	2.204(3)
V(3)-O(1W)	2.03	2.180(4)
O(7)-C(1)	1.30	1.433(7)
O(8)-C(7)	1.30	1.259(7)
O(9)-C(8)	1.30	1.325(5)
O(10)-C(14)	1.30	1.310(6)
O(11)-C(15)	1.30	1.336(5)
O(12)-C(21)	1.30	1.309(5)
Angles	Angle values (°)	Angle values (°)
O(1)-V(1)-O(6)	99.18	103.03(17)
O(1)-V(1)-O(4)	100.32	103.18(17)
O(6)-V(1)-O(4)	88.53	93.88(15)
O(1)-V(1)-O(7)	97.94	102.01(17)
O(6)-V(1)-O(7)	89.25	90.56(17
O(4)-V(1)-O(7)	161.73	152.69(16)
O(1)-V(1)-O(8)	95.30	100.36(18)
O(6)-V(1)-O(8)	165.30	156.20(15)
O(4)-V(1)-O(8)	86.40	84.93(17)
O(7)-V(1)-O(8)	91.25	80.33(19)
O(1)-V(1)-O(12)	168.78	176.19(17)
O(6)-V(1)-O(12)	70.13	74.47(12)
O(4)-V(1)-O(12)	83.23	78.40(12)
O(7)-V(1)-O(12)	78.96	76.89(13)
O(8)-V(1)-O(12)	95.55	82.04(13)
O(4)-V(2)-O(9)	172.33	153.53(15)
O(5)-V(2)-O(9)	81.44	88.57(14)
O(2)-V(2)-O(10)	97.42	102.7(2)
O(4)-V(2)-O(10)	88.15	86.67(15)

O(5)-V(2)-O(10)	156.85	152.79(15)
O(9)-V(2)-O(10)	86.30	79.36(14)
O(3)-V(3)-O(5)	103.61	102.16(19)
O(3)-V(3)-O(6)	109.38	105.34(17)
O(5)-V(3)-O(6)	91.93	92.03(14)
O(3)-V(3)-O(11)	92.71	95.80(16)
O(5)-V(3)-O(11)	93.33	100.14(14)
O(6)-V(3)-O(11)	155.37	152.82(14)
O(3)-V(3)-O(12)	173.73	172.09(16)
O(5)-V(3)-O(12)	82.37	83.65(14)
O(6)-V(3)-O(12)	71.88	79.86(13)
O(2)-V(2)-O(4)	94.34	103.26(18)
O(2)-V(2)-O(5)	102.52	103.6(2)
O(4)-V(2)-O(5)	101.45	94.01(15)
O(2)-V(2)-O(9)	91.93	101.69(17)
V(3)-O(12)-V(1)	97.01	84.98(10)
O(11)-V(3)-O(12)	85.00	77.54(12)
V(2)-O(4)-V(1)	112.20	125.61(18)
V(3)-O(5)-V(2)	119.40	120.39(17)
V(1)-O(6)-V(3)	120.79	119.22(17)

[a] Structure reported in Avecilla, F.; Geraldes, Carlos F. G. C.; Castro, M. Margarida C. A. A New Trinuclear Oxovanadium(V) Complex with DMPP Ligands − Synthesis and Structural Characterization in the Solid State and in Aqueous Solution. *Eur. J. Inorg. Chem*. **2001**, *2001*, 3135-3142. [b] Standard deviations in parenthesis.

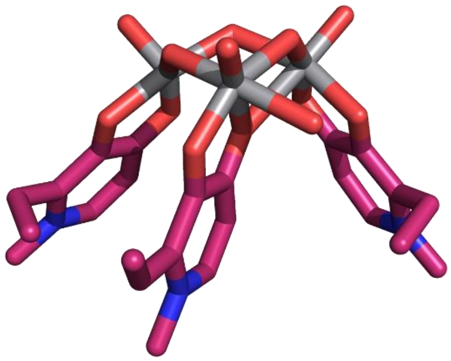

Figure S4. Structure of the trinuclear cluster $[V^{V}_3O_6(empp)_3(H_2O)]$ formed by empp(–) in the presence of HEWL in structure **A**. V atoms are in grey, empp(–) ligands are in hot pink, while oxygens are in red.

Table S3. Vanadium binding sites found in the crystal structures of HEWL treated with potential [$V^{IV}OL_2$] drugs and [$V^{IV}O(empp)_2$]. The ligands observed for each vanadium binding site is also reported. Values in parentheses correspond to the occupancy of the metal-containing fragment.

**HEWL–[$V^{IV}OL_2$] adduct**	**HEWL–$V^{IV}O(empp)_2$] (Structure A)**	**HEWL–[$V^{IV}O(empp)_2$] (Structure B)**	**HEWL–[$V^{IV}O(empp)_2$] (Structure C)**	**HEWL–[$V^{IV}O(malt)_2$] (Structure A)**[a]	**HEWL–[$V^{IV}O(malt)_2$] (Structure A')**[a]	**HEWL–[$V^{IV}O(malt)_2$] (Structure B)**[a]	**HEWL–[$V^{IV}O(pic)_2(H_2O)$]**[b]	**HEWL–[$V^{IV}O(phen)_2$]**[c]	**HEWL–[$V^{IV}O(bipy)_2$]**[c]
PDB code	8OM8	8OMS	8OMT	8AJ3	8AJ4	8AJ5	4C3W	7Q0V	7Q0U
**V binding site**									
Glu35	-	-	-	-	-	$V^{IV}O$ (0.35)<br>3 H_2O	-	-	-
Asn46 and Asp52	-	-	-	-	-	-	-	$V^{IV}O$ (0.75)<br>phen<br>H_2O	$V^{IV}O$ (0.90)<br>bipy<br>H_2O
Asp48	$V^{IV}O$ (0.60)<br>empp<br>H_2O	-	-	-	-	$V^{IV}O$ (0.50)<br>3 H_2O	-	-	-
Asp52	-	-	-	-	-	-	$V^{IV}O$ (0.65)<br>2 pic	-	-
Asn65	-	-	-	$V^{IV}O$ (0.50)<br>4 H_2O	$V^{IV}O$ (0.30)<br>2 malt	-	-	-	-
Asp87	-	-	-	-	-	$V^{IV}O$ (0.50)<br>3 H_2O	-	-	-
Asp101	-	-	-	-	-	-	-	$V^{V}O_2$ (0.50)	-
Asp119	-	-	-	-	-	$V^{IV}O$ (1.00)	-	-	-
C-terminal carboxylate	-	-	-	-	-	V (0.25)<br>3 H_2O	-	-	-
**Non-covalent binding**									

Arg5, Cys6, Glu7	-	$V^{IV}O$ (0.40) 2 empp H_2O	$V^{IV}O$ (0.80) 2 empp H_2O	$V^{IV}O$ (0.70) 2 malt H_2O	$V^{IV}O$ (0.80) 2 malt H_2O	-	-	-	-
Arg5, Lys33	3 $V^{V}O_2$ (0.60) 3 empp H_2O	-	-	-	-	-	-	-	-
Arg73, Arg74	-	-	-	$V^{IV}O$ (0.30) malt 3 H_2O	$V^{IV}O$ (0.30) 2 malt H_2O	-	-	-	-
Arg125	-	-	$V^{IV}O$ (0.40) empp 2 H_2O	-	-	-	-	-	-
Asp119, Gln121	-	-	-	-	-	-	-	$V^{IV}O$ (0.50)	-
	-	-	-	-	-	-	-	$V^{V}O_2$ (0.50)	-

[a] Structure reported in Ferraro, G.; Paolillo, M.; Sciortino, G.; Garribba, E.; Merlino, A. Multiple and Variable Binding of Pharmacologically Active Bis(maltolato)oxidovanadium(IV) to Lysozyme. *Inorg. Chem.* **2022**, *61*, 16458-16467; [b] Structure reported in Santos, M. F. A.; Correia, I.; Oliveira, A. R.; Garribba, E.; Costa Pessoa, J.; Santos-Silva, T. Vanadium Complexes as Prospective Therapeutics: Structural Characterization of a V^{IV} Lysozyme Adduct. *Eur. J. Inorg. Chem.* **2014**, 3293-3297; [c] Structure reported in Santos, M. F. A.; Sciortino, G.; Correia, I.; Fernandes, A. C. P.; Santos-Silva, T.; Pisanu, F.; Garribba, E.; Costa Pessoa, J. Binding of $V^{IV}O^{2+}$, $V^{IV}OL$, $V^{IV}OL_2$ and $V^{V}O_2L$ Moieties to Proteins: X-ray/Theoretical Characterization and Biological Implications. *Chem. Eur. J.* **2022**, e202200105.
